# Cesarean Delivery in Women With Genital Herpes in Washington State, 1989–1991

**DOI:** 10.1155/S1064744997000082

**Published:** 1997

**Authors:** Jeanne M. Marrazzo, Grace C. John, Marijane A. Krohn, Lawrence Corey

**Affiliations:** 1 Department of Medicine University of Washington Seattle WA USA; 2 Department of Epidemiology University of Washington Seattle WA USA; 3 Department of Laboratory Medicine University of Washington Seattle WA USA; 4 Department of Microbiology University of Washington Seattle WA USA; 5 Broadway STD Clinic 1001 Broadway Suite 320 Mailstop 359928 Seattle WA 98122 USA

## Abstract

*Objective:* The purpose of this study was to determine whether the proportion of cesarean deliveries in pregnant women with a history of genital herpes and no active lesions at birth is higher than that in women with no history of genital herpes, and to determine whether this risk was modified by birth facilities' underlying prevalence of cesarean delivery.

*Methods:* This was a retrospective survey. Women who gave birth in Washington state from 1989 to 1991 were identified from the state birth records and were classified as having clinical genital herpes during pregnancy (N = 1,094) or history of genital herpes only (N = 4,163) at delivery. Women without genital herpes (N = 5,257) were randomly selected from remaining births.

*Results:* The main outcome measure was primary cesarean delivery, excluding those performed for indications other than genital herpes. Prevalence of primary cesarean delivery was 59.5% in women with clinical herpes during pregnancy and 12.5% in women with history of herpes, both significantly different from prevalence of 11.2% in unexposed women. Age-adjusted risk for cesarean delivery among women with a history of herpes was 1.13 [95% confidence interval (CI): 0.93, 1.37]. When baseline cesarean delivery prevalence was above 20%, this risk was 1.2 (95% CI: 1.0, 1.4; *P* = 0.058), compared to 1.1 (95% CI: 0.9, 1.3; *P* = 0.186) where cesarean delivery prevalence was below 20%.

*Conclusions:* Women with history of genital herpes appear to have a slightly elevated risk of cesarean delivery, particularly in hospital settings with baseline prevalence of primary cesarean delivery above 20%. This rate is somewhat lower than that noted in a previous survey, suggesting that practitioners are following standard guidelines. Evaluations of cesarean delivery for genital herpes in other states should be performed.


Infectious Diseases in Obstetrics and Gynecology 5:29–35 (1997)
© 1997 Wiley-Liss, Inc.

### Cesarean Delivery in Women With Genital Herpes in Washington State, 1989–1991

Jeanne M. Marrazzo,[1*] Grace C. John,[1] Marijane A. Krohn,[2] and Lawrence Corey[1,3,4]

[1]*Department of Medicine, University of Washington, Seattle, WA*
[2]*Department of Epidemiology, University of Washington, Seattle, WA*
[3]*Department of Laboratory Medicine, University of Washington, Seattle, WA*
[4]*Department of Microbiology, University of Washington, Seattle, WA*

### Abstract

***Objective:*** The purpose of this study was to determine whether the proportion of cesarean deliveries in pregnant women with a history of genital herpes and no active lesions at birth is higher than that in women with no history of genital herpes, and to determine whether this risk was modified by birth facilities' underlying prevalence of cesarean delivery.

***Methods:*** This was a retrospective survey. Women who gave birth in Washington state from 1989 to 1991 were identified from the state birth records and were classified as having clinical genital herpes during pregnancy (N = 1,094) or history of genital herpes only (N = 4,163) at delivery. Women without genital herpes (N = 5,257) were randomly selected from remaining births.

***Results:*** The main outcome measure was primary cesarean delivery, excluding those performed for indications other than genital herpes. Prevalence of primary cesarean delivery was 59.5% in women with clinical herpes during pregnancy and 12.5% in women with history of herpes, both significantly different from prevalence of 11.2% in unexposed women. Age-adjusted risk for cesarean delivery among women with a history of herpes was 1.13 [95% confidence interval (CI): 0.93, 1.37]. When baseline cesarean delivery prevalence was above 20%, this risk was 1.2 (95% CI: 1.0, 1.4; $P = 0.058$), compared to 1.1 (95% CI: 0.9, 1.3; $P = 0.186$) where cesarean delivery prevalence was below 20%.

***Conclusions:*** Women with history of genital herpes appear to have a slightly elevated risk of cesarean delivery, particularly in hospital settings with baseline prevalence of primary cesarean delivery above 20%. This rate is somewhat lower than that noted in a previous survey, suggesting that practitioners are following standard guidelines. Evaluations of cesarean delivery for genital herpes in other states should be performed. Infect. Dis. Obstet. Gynecol. 5:29–35, 1997.
© 1997 Wiley-Liss, Inc.

Key words
herpes simplex virus; cesarean delivery; neonatal herpes; birth certificates; Washington state

Standard obstetric practice recommends cesarean section as the preferred method of delivery for women with genital herpes (GH) clinically evident at the time of delivery.[9,35] The consequences of neonatal acquisition of herpes simplex virus (HSV) are frequently severe, ranging from permanent neurological impairment to death.[2,3,34] Accordingly, the proportion of cesarean deliveries per-

Contract grant sponsor: NIAID STD/AIDS Research; Contract grant number: AI-07140; Contract grant sponsor: NIH; Contract grant number: AI-30731.
*Correspondence to: Dr. Jeanne M. Marrazzo, Broadway STD Clinic, 1001 Broadway, Suite 320, Mailstop 359928, Seattle, WA 98122. E-mail: jmm2@u.washington.edu

Received 27 December 1996
Accepted 16 May 1997

formed in women with GH evident at delivery is high. However, less is known about rates of cesarean delivery in women who report a *history* of GH, but who do not evidence lesions at delivery. Current guidelines advise against cesarean delivery in this group,[9,16,17,20,21] since the likelihood of viral shedding in genital secretions at delivery in the absence of lesions is small (0.6–1.4%), as is the attack rate for neonatal infection even in the presence of asymptomatic viral shedding (2–5%).[8,11,12,22] Cesarean delivery has also failed to prevent neonatal HSV acquisition in some cases, possibly as a consequence of prior in utero infection.[3,5]

Despite current recommendations, practitioners may perform cesarean delivery in pregnant women with a history of GH and no clinically evident lesions for several reasons.[30,31] First, neither antepartum cultures, clinical diagnosis at delivery, nor history of symptomatic GH during the pregnancy accurately predicts asymptomatic viral shedding at vaginal delivery.[13,18,19,23,28] Less than one third of women who transmit HSV to neonates report a recurrence during the current pregnancy.[5,14,15] Most neonates who acquire HSV at vaginal delivery do so in the absence of clinically evident infection, often in the setting of recently acquired maternal GH.[10,27] Second, the severity of neonatal disease when it does occur is considerable, incurring an estimated 25% mortality rate[3] and cumulative economic costs of over $500 million annually[37] (an excess of $25,000/case for initial hospital costs alone[33]). Finally, the incidence of cesarean delivery varies by clinical and geographic setting[24,25]; individual practitioners' tendencies to perform cesarean delivery in the setting of a prior history of GH may be influenced by this "baseline" rate.

We addressed the hypothesis that despite current clinical recommendations, the proportion of cesarean deliveries in pregnant women with a history of genital herpes and no active lesions at birth is higher than that in women who give no history of prior genital herpes. We also examined whether this proportion was modified by the baseline incidence of cesarean delivery in the health care facilities studied.

### SUBJECTS AND METHODS

### Study Design and Population

The study employed a retrospective cohort design. Subjects were selected from the singleton birth records of Washington state from the years 1989 through 1991. The exposed groups were defined by the presence of the following under "Medical Pregnancy Complications": 1) women diagnosed with "genital herpes (active)" (N = 1,094) and 2) women diagnosed with "genital herpes (history)" (N = 4,163). The nonexposed group (N = 5,257) was randomly selected from the pool of remaining total singleton births over the same 3 years.

"Active genital herpes" on the Washington state birth certificate refers to the presence of visible lesions at delivery, *or* to a symptomatic episode occurring at any time during that pregnancy as reported by the patient; consequently, we renamed this designation "clinical herpes during pregnancy" for the purposes of our study. "History of genital herpes" refers to any history of genital herpes prior to the current pregnancy as reported by the patient.

### Calculation of Risk Estimates

The outcome used in the calculation of risk estimates was primary cesarean delivery. Repeated cesarean delivery, with or without a trial of labor, was not included as an outcome, because these cesarean deliveries were generally for an indication other than genital herpes. To confirm that the choice of primary cesarean delivery as the outcome of interest accurately reflected risk, and that the exclusion of repeat cesarean delivery did not introduce confounding, two analyses were performed. The first analysis defined the outcome event as primary cesarean delivery only, excluding repeat cesarean delivery. The second analysis included all cesarean deliveries (repeat and primary) as outcome events. In both analyses, potential indications for cesarean delivery other than herpes were excluded. These conditions included prolonged or dysfunctional labor, breech/malpresentation, cephalopelvic disproportion, eclampsia, maternal fever, placenta previa, abruptio placentae, and fetal distress. If evidence of any of these indications was noted on the birth certificate, the record was quantified and excluded from the calculation of risk estimates, so that the estimate of excess cesarean delivery would more closely reflect that due to genital herpes status alone.

Methods of delivery were determined for each group and classified as vaginal or cesarean delivery (primary or repeat). Risk of cesarean delivery for both groups of women with herpes (clinical herpes during pregnancy or history of herpes prior to current pregnancy) relative to women without herpes was determined. The following factors in modifying and confounding risk estimates were assessed: age, alcohol and cigarette use during pregnancy, race, marital status, urban vs. rural residence, annual income, and nulliparity. Selected labor and delivery complications (fever, premature rupture of membranes) and adverse neonatal outcomes (prematurity, anemia, sepsis) were compared across the three groups.

To determine whether the baseline incidence of cesarean delivery in a given health care facility had any effect on the frequency of cesarean delivery for GH, a subanalysis stratifying by facilities' prevalence of cesarean delivery was done. The percentage of cesarean deliveries in exposed and nonexposed groups was determined for 82 acute care facilities for the study period (1989 through 1991). The facilities represented a wide intrastate geographic range. They were divided into two groups: those with a prevalence of cesarean delivery above 20% (N = 34), and those with a prevalence below 20% (N = 48). The stratum-specific relative risks for cesarean delivery were then calculated.

### Statistical Analysis

Statistical analysis was performed using SAS (SAS Institute, Cary, NC). Adjusted risk estimates were obtained using Epi-Info (Centers for Disease Control and Prevention, Atlanta, GA). Differences between the exposed and nonexposed groups were assessed using chi-square statistics and are reported significant at the two-tailed $P < 0.05$ level and adjusted for potential confounders as noted.

### RESULTS

### Demographics

Table 1 summarizes the descriptive characteristics of women with and without GH. Small but statistically significant differences existed with regard to age, race, marital status, urban vs. rural residence, alcohol use during pregnancy, and annual income. No significant differences were noted with regard to parity or smoking during pregnancy.

### Prevalence of Cesarean Delivery

The prevalence of primary cesarean delivery was 61.7% in women with clinical herpes during pregnancy, and 13.3% in women with history of herpes prior to the current pregnancy. Both differed significantly from the prevalence of 11.9% seen in unexposed women (Table 2).

Major indications for primary cesarean delivery in the nonexposed women included cephalopelvic disproportion (30.34%), breech presentation (21.69%), and dysfunctional labor (9.83%). Placental problems (previa or abruptio), maternal fever, fetal distress, and eclampsia accounted for relatively few such births. A total of 70.85% of the primary cesarean deliveries were accounted for by the seven indications listed. Only 19.25% of the repeat cesarean deliveries were accounted for by these indications, consistent with the fact that many repeat sections are performed for the indica-

**TABLE 1. Characteristics of study subjects by GH status [N (%)]‡**

| | Clinical GH during pregnancy (N = 1,094) | History of GH (N = 4,163) | No GH (N = 5,257) |
|---|---|---|---|
| Age (years) | | | |
| ≤18 | 39 (3.96) | 61 (1.59) | 195 (3.85) |
| 19–29 | 568 (56.1)* | 1,941 (50.6)* | 3,093 (62.8) |
| ≥30 | 403 (39.8)* | 1,827 (47.6)* | 1,632 (33.2) |
| Race | | | |
| White | 937 (85.7)* | 3,642 (87.5)* | 4,263 (81.1) |
| Black | 50 (4.6)* | 162 (3.9)* | 148 (3) |
| Native American | 31 (2.8) | 79 (1.9)* | 133 (2.5) |
| Asian | 22 (2)* | 86 (2)* | 271 (5.2) |
| Hispanic | 26 (2.4)* | 97 (2.3)* | 358 (7) |
| Marital status | | | |
| Single | 802 (73.3) | 3,264 (78.4)* | 3,979 (75.7) |
| Married | 292 (26.7) | 893 (21.6)* | 1,264 (24.3) |
| Prior pregnancy | | | |
| No | 566 (51.7) | 1,804 (43.3) | 2,202 (41.9) |
| Yes | 528 (48.3) | 2,359 (56.7) | 3,050 (58.1) |
| Residence | | | |
| Urban | 612 (56.4)* | 2,215 (53.8) | 2,825 (54.3) |
| Rural | 474 (43.6) | 1,904 (46.2) | 2,375 (45.7) |
| Alcohol use during pregnancy | | | |
| Yes | 92 (8.4)* | 330 (7.9)* | 239 (4.6) |
| No | 931 (85.1) | 3,603 (86.5) | 4,570 (86.9) |
| Smoking during pregnancy | | | |
| Yes | 216 (19.7) | 852 (20.5) | 1,042 (19.8) |
| No | 834 (76.2) | 3,139 (75.4) | 3,870 (73.6) |
| Annual income | | | |
| <15K | 92 (14.6) | 351 (14.2) | 414 (15.1) |
| 15–25K | 100 (15.9) | 367 (14.9)* | 490 (17.9) |
| >25–45K | 354 (56.2) | 1,365 (55.3) | 1,496 (54.8) |
| >45K | 8 (13.3) | 384 (15.6)* | 331 (12.1) |

*$P < 0.05$.
‡Subgroup totals may not equal N at top of column because data were not complete for all subjects on all variables.

TABLE 2. Proportion of cesarean deliveries by GH status, stratified by cesarean section (CS) prevalence of birth facility

| | Clinical GH during pregnancy | | History of GH | | No GH | |
|---|---|---|---|---|---|---|
| | N | % | N | % | N | % |
| | *All Birth Facilities Combined* | | | | | |
| Primary CS | 557 | 61.7 | 437 | 13.3 | 481 | 11.9 |
| Repeat CS[a] | 104 | 11.5 | 220 | 6.7 | 225 | 5.6 |
| Vaginal delivery | 241 | 26.7 | 2,638 | 8.0 | 3,333 | 82.5 |
| Total | 902 | | 3,295 | | 4,039 | |
| | *Birth Facilities With CS Prevalence >20%* | | | | | |
| Primary CS | 261 | 71.3 | 189 | 16.2 | 231 | 13.8 |
| Repeat CS[a] | 40 | 10.9 | 86 | 7.4 | 111 | 6.6 |
| Vaginal delivery | 65 | 17.8 | 892 | 76.4 | 1,336 | 79.6 |
| Total | 366 | | 1,167 | | 1,678 | |
| | *Birth Facilities With CS Prevalence <20%* | | | | | |
| Primary CS | 296 | 55.2 | 248 | 11.6 | 250 | 10.6 |
| Repeat CS[a] | 64 | 11.9 | 134 | 6.3 | 114 | 4.8 |
| Vaginal delivery | 176 | 32.8 | 1,746 | 82.0 | 1,997 | 84.6 |
| Total | 536 | | 2,128 | | 2,361 | |

[a]Excludes repeat CS with trial of labor.

tion of having had a prior cesarean delivery. As would be expected, indications for cesarean delivery other than herpes in the exposed groups were similar to those described for the nonexposed group (Fig. 1).

After exclusion of births in which non-GH conditions could have necessitated cesarean delivery, 3,382 subjects remained in the group without GH, 952 with clinical GH during pregnancy, and 4,535 with a history of herpes prior to the current pregnancy. Regardless of inclusion or exclusion of cesarean delivery for other indications, the age-adjusted relative risk for cesarean delivery among women with a history of herpes was 1.13 [95% confidence interval (CI): 0.93, 1.37]. This risk also did not change when repeat cesarean delivery was included as an outcome event. In all analyses, no significant confounding was detected for age, race, alcohol intake, smoking, marital status, rural or urban residence, or income.

The relative risk of cesarean section for women with clinical herpes during pregnancy, compared to women without herpes, was 12.88 (95% CI: 11.24, 14.77).

### Analysis by Birth Facilities' Cesarean Prevalence

Results of the analysis stratified for birth facility are shown in Table 2. The risk for cesarean delivery in women with a history of herpes was 1.2 (95% CI: 1.0, 1.4; $P = 0.058$) in the group of birth facilities with baseline cesarean delivery prevalence above 20%, compared to 1.1 (95% CI: 0.9, 1.3; $P = 0.186$) in that with cesarean delivery prevalence below 20%.

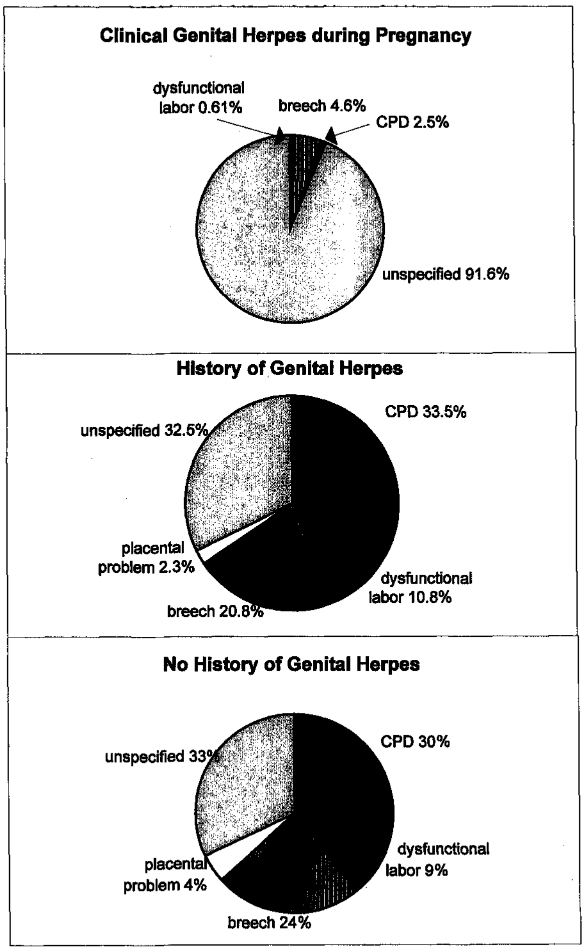

Fig. 1. Major indications for cesarean delivery. CPD = cephalopelvic disproportion.

### Adverse Maternal and Fetal Outcomes

Associations between the occurrence of adverse maternal and fetal outcomes and maternal herpes status were not significant for neonatal sepsis or anemia in either of the exposed groups. Neither were women with historical herpes more likely to have preterm labor (data not shown).

### DISCUSSION

Neonatal herpes is associated with significant morbidity and mortality.[1–5,34] Its incidence in the

United States is thought to be increasing, with current estimates between 400 and 1,000 cases/year.[6,36] This increase may be associated with the finding that more women of childbearing age are seeking evaluation for genital herpes.[7] Of the two herpes virus types, HSV-2 is responsible for the majority of genital infection, while HSV-1 accounts for the remainder. The prevalence of seropositivity to HSV-2 in the general U.S. population in 1990 has been estimated at 21.7%, an increase of 32.3% over the past decade.[32] More recent evidence suggests that this increase may be continuing. In a recent population-based survey of 1,770 inner-city residents in San Francisco, seroprevalence to HSV-2 was 33% overall, with peaks in those 30–34 years of age (49%) and the highest prevalence seen in black women (55%). HSV-2 seropositivity in women was 41% overall.[38] Seroprevalence among pregnant women was not assessed in that study; however, earlier surveys of pregnant women have indicated that 20–30% show immunologic evidence of prior HSV-2 infection.[8]

Cesarean sections are a commonly performed procedure in current obstetric practice in the United States, accounting for 15–30% of deliveries, depending on the geographic area sampled. There is some regional variation in cesarean section rate; the Western region of the United States had estimated rates of 19.8% (total) and 15.1% (primary) which are lower than the national average.[25] The determination of optimal method of delivery is influenced by a variety of factors in addition to the conventional "pathologic" indications. Hospital size, patient insurance status, and practitioner preference may contribute to decision-making in this regard. Aggregate financial cost and maternal morbidity are considerably higher for cesarean sections than for vaginal deliveries.[29]

Our primary group of interest was women who had only a history of genital herpes but no symptoms throughout pregnancy or at delivery. These women had a slightly elevated percentage of births by cesarean section (12.5%) compared with women who had no history of herpes (11.2%). This finding may reflect appropriate compliance with current guidelines; however, this risk estimate still approximates a 13% increase in cesarean delivery in exposed women above those with no herpes history. Such an elevation could, in a large number of women, account for significant, and potentially avoidable, maternal and infant morbidity and economic cost. This may be particularly true in populations in which section rates are generally higher than those seen in our study. This concept is, in fact, borne out by the more significant risk of cesarean delivery for women with historical herpes who gave birth in facilities with a higher baseline frequency of cesarean delivery (Table 2). A more refined analysis than was possible with the present data set, controlling for potential confounders of this relationship (such as the facilities' tertiary referral base and high-risk obstetric practice), might prove illuminating.

A retrospective cohort analysis performed in 1984 also used the Washington State Vital Records to identify 1,156 women with genital herpes in King, Snohomish, and Pierce Counties during 1980 through 1983 (Wolf and Corey, unpublished data).[26] The definition of "herpes" was the same as that in the 1989–1991 cohort; however, "active" and "history" herpes status were not distinguished on the birth record at that time. A hospital chart review of 909 of these births revealed that the prevalence of primary cesarean delivery in women with active herpes at delivery was 60%—very similar to that in our study. Prevalence of cesarean delivery in women with a history of herpes was 18% in the 1984 chart review, compared to our prevalence of 12.5%. Prevalence of cesarean delivery in women with no herpes in 1980–1983 was 10.3%, relatively close to our finding of 11.2%. These data suggest that the prevalence of excess cesarean delivery in women with a history of genital herpes has declined over the past decade, while that in women with active HSV has remained constant. The similar cesarean section rate in the 1980–1983 cohort, in which chart reviews were performed to authenticate reports on the birth records, supports the accuracy of our data.

In our study, 59% of women with active herpes underwent cesarean section. This surprisingly low rate requires explanation. It is probably due to the fact that the definition of active herpes in Washington state includes active lesions *at any time during* pregnancy; accordingly, not all cases coded as "active" would require CS if the symptomatic episode had occurred well prior to delivery.

Interpretation of our results should be viewed in light of the study's limitations. First, the definition of active herpes included symptoms anytime during pregnancy; therefore, this characterization is only a crude approximation of herpes which is truly active at the time of delivery. Nonetheless, the high proportion of cesarean delivery with indication unspecified in this group supports the hypothesis that active herpes at delivery figured prominently in choice of cesarean section as delivery method. Second, the birth record does not allow for the distinction among herpes that is diagnosed 1) by recognition of visible lesions by the physician, 2) by culture documentation, or 3) by patient report. Historical herpes may most often be a diagnosis provided by the patient and while specific, is very insensitive, for the presence of GH; active GH, in this setting, could fall into any of the three categories. Misclassification of exposure status could have arisen from this lack of distinction, and could bias the risk estimates for cesarean delivery in either direction. Third, the decision to proceed with cesarean section is informed by a variety of factors such as obstetric history, patient and/or practitioner preference, clinical status of infant and mother, and regional tendencies. The structure of the Washington state birth record allowed only for the inferred indication for cesarean delivery in our study population. Consequently, a complex analysis of the role of GH in this decision-making process was not possible with our data set. Finally, use of birth certificate data itself presents a limitation, in that coding of the method of delivery may vary from one hospital to another, and is probably not as accurate as data obtained directly from computerized hospital discharge data.[39] An analysis using the latter to assess our study's findings would be useful.

In summary, our study is the first assessment specifically directed at estimating the frequency of cesarean section in women who report a history of GH but who do not evidence active lesions at the time of delivery. We found a slightly elevated risk of cesarean delivery among women with a history of GH, which may be modified by the underlying rate of this procedure in the birth facility involved. Further evaluations—both retrospective and prospective—of this relationship in other populations could provide a stronger basis for refining recommendations, reducing costs, and minimizing maternal and neonatal morbidity.

### ACKNOWLEDGMENTS

The authors gratefully acknowledge the insight and assistance of Dr. Anna Wald in reviewing this manuscript.

### REFERENCES

1. Whitley RJ, Nahmias AJ, Visintine AM, Fleming CL, Alford CA: The natural history of herpes simplex virus infection of the mother and newborn. Pediatrics 66:489–494, 1980.
2. Whitley R, Arvin A, Prober C, et al.: Predictors of morbidity and mortality in neonates with herpes simplex virus infections. N Engl J Med 324:450–454, 1991.
3. Chang TY: Neonatal herpes: Incidence, prevention and consequences. Am J Prev Med 4:47–53, 1988.
4. Jenkins M, Kohl S: New aspects of neonatal herpes. Infect Dis Clin North Am 6:57–74, 1992.
5. Whitley RJ, Corey L, Arvin A, et al.: Changing presentation of herpes simplex virus infection in neonates. J Infect Dis 158:109–116, 1988.
6. Sullivan-Bolyai J, Hull HF, Wilson C, Corey L: Neonatal herpes simplex virus infection in King County, Washington. Increasing incidence and epidemiologic correlates. JAMA 250:3059–3062, 1983.
7. Becker CG, Blount JH, Guinan MG: Genital herpes infections in private practice in the United States 1966–1981. JAMA 253:1601–1603, 1985.
8. Prober CG, Arvin AM: Genital herpes and the pregnant woman. In Remington JS, Schwartz MN (eds): Current Clinical Topics in Infectious Diseases. Cambridge, MA: Blackwell, pp 1–26, 1989.
9. Prober CG, Corey L, Brown ZA, Hensleigh PA, et al.: The management of pregnancies complicated by genital infections with herpes simplex virus. Clin Infect Dis 15:1031–1038, 1992.
10. Brown ZA, Benedetti J, Ashley R, Burchett S, et al.: Neonatal herpes simplex virus infection in relation to asymptomatic maternal infection at the time of labor. N Engl J Med 324:1247–1252, 1991.
11. Prober CG, Sullender W, Yasukawa LL, et al.: Low risk of herpes simplex infections in neonates exposed to virus at the time of vaginal delivery to mothers with recurrent genital herpes simplex virus infections. N Engl J Med 316:240–244, 1987.
12. Harger JH, Pazin GJ, Armstrong JA, et al.: Characteristics and management of pregnancy in women with genital herpes simplex virus infection. Am J Obstet Gynecol 145:784–791, 1983.
13. Prober CG, Hensleigh PA, Boucher FD, et al.: Use of routine viral cultures at delivery to identify neonates exposed to herpes simplex virus. N Engl J Med 318: 887–891, 1988.
14. Harger JH, Meyer MP, Amortegui AJ: Changes in the frequency of genital herpes recurrences as a function of time. Obstet Gynecol 67:637–642, 1986.
15. Prober CG, Sullender WM, Yasukawa LL, et al.: Low risk of herpes simplex virus infections in neonates exposed to virus at the time of vaginal delivery to mothers with recurrent genital herpes simplex virus infections. N Engl J Med 316:240–244, 1986.

16. Sperling RS, Berkowitz RL: Obstetric management of women with a history of recurrent genital herpes. Am J Perinatol 6:275–277, 1989.
17. Gibbs RS, Mead PB. Editorial: Preventing neonatal herpes—Current strategies. N Engl J Med 326:946–947, 1992.
18. Catalano PM, Merritt AO, Mead PB: Incidence of genital herpes simplex virus at the time of delivery in women with known risk factors. Am J Obstet Gynecol 164:1303–1306, 1991.
19. Arvin AM, Hensleigh PA, Prober CG, et al.: Failure of antepartum maternal cultures to predict the infant's risk of exposure to herpes simplex virus at delivery. N Engl J Med 316:796–800, 1986.
20. Libman MD, Dascal A, Kramer MS, Mendelson J: Strategies for the prevention of neonatal infection with herpes simplex virus: A decision analysis. Rev Infect Dis 13:1093–1104, 1991.
21. Forsgren M, Sterner G, Anz'en B, Enocksson E, et al.: Management of women at term with pregnancy complicated by herpes simplex. Scand J Infect Dis 71:58–66, 1990.
22. Boucher FD, Yasukawa LL, Bronzan RN, et al.: A prospective evaluation of primary genital herpes simplex virus type 2 infections acquired during pregnancy. Pediatr Infect Dis J 9:499–504, 1990.
23. Wittek AE, Yeager AS, Au DS, Hensleigh PA: Asymptomatic shedding of herpes simplex virus from the cervix and lesion site during pregnancy. Am J Dis Child 138:439–442, 1984.
24. Porreco RP: High cesarean section rate: A new perspective. Obstet Gynecol 65:307–311, 1985.
25. Centers for Disease Control and Prevention: Rates of cesarean delivery—United States, 1991. MMWR 42: 285–289, 1993.
26. Wolf M, Tsu V, Daling J: Genital herpes and pregnancy outcome: A population-based cohort study. Am J Epidemiol 120:485–486, 1984.
27. Brown ZA, Vontver LA, Benedetti J, et al.: Effects on infants of a first episode of genital herpes during pregnancy. N Engl J Med 317:1246–1251, 1987.
28. Brown ZA, Berry SB, Vontver LA: Genital herpes simplex virus infections complicating pregnancy. Natural history and peripartum management. J Reprod Med 31: 420–425, 1986.
29. Feldman GB, Freiman JA. Prophylactic cesarean section at term? N Engl J Med 312:1264–1267, 1985.
30. Yeager AS, Arvin AM: Reasons for the absence of a history of recurrent genital infections in mothers of neonates infected with herpes simplex virus. Pediatrics 73: 188–192, 1984.
31. Harger JH, Amortegui AJ, Meyer MP, Pazin GJ: Characteristics of recurrent genital herpes simplex infections in pregnant women. Obstet Gynecol 73:367–372, 1989.
32. Johnson R, Lee F, Hadgu A, McQuillan G, Keesling S, Nahmias A: U.S. genital herpes trends during the first decade of AIDS—Prevalences increased in young whites and elevated in blacks. In: Program and Abstracts of the 10th International Meeting of the International Society of STD Research, Helsinki, Finland, August 29–September 1, 1993, Abstract 22.
33. Randolph AG, Washington AE, Prober CG: Cesarean delivery for women presenting with genital herpes lesions. JAMA 270:77–82, 1993.
34. Whitley RJ: Herpes simplex virus infections. In Remington JS, Klein JO (eds): Infectious Diseases of the Fetus and Newborn Infant. 3rd ed. Philadelphia: W.B. Saunders, pp 283–305, 1990.
35. Gibbs RS, Amstey MS, Sweet RL, et al.: Management of genital herpes infection in pregnancy. Obstet Gynecol 71:779–780, 1988.
36. Stone KM, Brooks CA, Guinnan ME, et al.: National surveillance for neonatal herpes simplex virus infections. Sex Transm Dis 16:152, 1989.
37. Prober CG: Reducing the risk of perinatal transmission of herpes simplex virus type 2. Infect Med 10(3):21–24, 27–28, 44, 1993.
38. Siegel D, Golden E, Washington AE, et al.: Prevalence and correlates of herpes simplex infections. The population-based AIDS in multiethnic neighborhoods study. JAMA 268:1702–1708, 1992.
39. Parrish KM, Holt VL, Connell FA, Williams B, LoGerfo JP: Variations in the accuracy of obstetric procedures and diagnoses on birth records in Washington state, 1989. Am J Epidemiol 138:119–127, 1993.
